# Supplementary material for: Vulnerabilities of Rohingya and host community children in Bangladesh: A qualitative study on child labor, well-being, and the impact of COVID-19
Source: PLOS Glob Public Health. 2025 Jul 3;5(7):e0004865. doi: 10.1371/journal.pgph.0004865 (PMC12225819; doi:10.1371/journal.pgph.0004865)
Supplement: S1 Text — (PDF) [file pgph.0004865.s001.pdf]

Questionnaire for

Evaluation of Child Labor Situation in The Context of Rohingya And Host Community in Cox's Bazar, Bangladesh

☐ **Camp** **Age:**

☐ **Host** **Gender:**

- **How long have you been working here?**
  তুমি এখানে কবে থেকে কাজ করছো?

▪

- **Who inspired you to work here?**
  কে তোমাকে এখানে এসে কাজ করতে উৎসাহ দিয়েছে?

▪
▪
▪

- **Why did you start working outside home? (if confused, ask if any type of incidence/accident/ fire incident/family member death/ natural disaster/ pandemic pushed to start working?)**
  তুমি কেন ঘরের বাইরে কাজ করা শুরু করেছো? (বুঝতে বা বোঝাতে অসুবিধা হলে- কোন ধরনের ঘটনা/ দুর্ঘটনা/ অগ্নিকান্ডের ঘটনা/ পরিবারের সদস্যের মৃত্যু/ প্রাকৃতিক দুর্যোগ/ মহামারী ইত্যাদি এর পর থেকে সে ঘরের বাইরে কাজ করা শুরু করেছে কিনা, তা জিজ্ঞেস করুন?)

▪
▪
▪
▪
▪
▪

- **Why don't you go to school?**
  তুমি কেন স্কুলে যাও না?

▪
▪
▪
▪
▪
▪

- **What problems do you face working here?**
  তোমার কি এখানে কাজ করতে কোন অসুবিধা হয়?

▪
▪
▪
▪
▪
▪

- Have you experienced any injuries or health problems due to your work?
  তোমার কাজের কারণে তুমি কি কোনো আঘাত বা স্বাস্থ্য সমস্যার সম্মুখীন হয়েছে?
